# Serum Renin Levels Increase With Age in Boys Resulting in Higher Renin Levels in Young Men Compared to Young Women, and Soluble Angiotensin-Converting Enzyme 2 Correlates With Renin and Body Mass Index

**DOI:** 10.3389/fphys.2020.622179

**Published:** 2021-01-15

**Authors:** Lars Jehpsson, Jiangming Sun, Peter M. Nilsson, Andreas Edsfeldt, Per Swärd

**Affiliations:** ^1^Clinical and Molecular Osteoporosis Research Unit, Departments of Orthopedics and Clinical Sciences, Skåne University Hospital, Lund University, Malmö, Sweden; ^2^Department of Cardiovascular Research-Translational Studies and Cardiology, Skåne University Hospital, Lund University, Malmö, Sweden; ^3^Internal Medicine–Epidemiology, Department of Clinical Sciences, Skåne University Hospital, Lund University, Malmö, Sweden; ^4^Wallenberg Center for Molecular Medicine, Lund University, Malmö, Sweden; ^5^Department of Cardiology, Skåne University Hospital, Malmö, Sweden

**Keywords:** angiotensin-converting enzyme 2, body mass index, coronavirus–COVID-19, renin, renin–angiotensin–aldosterone system, severe acute respiratory coronavirus 2

## Abstract

**Background:** Age, sex, and body constitution may affect the shedding of membrane bound angiotensin-converting enzyme 2 (mACE2) and lead to a relative mACE2 deficiency. However, it is unclear if differences, reflected by serum renin levels, exist in the basal renin-angiotensin-system (RAS) between children and adults, boys, and girls as well as young women and young men. Furthermore, it remains to be investigated if renin and soluble ACE2 (sACE2) levels are correlated with body mass index (BMI) in children and young adults. The aim of this observational study was to assess age-and sex differences in serum renin, and the relationship between renin, soluble angiotensin-converting enzyme 2, and body mass index in a prospectively followed population-based cohort of children which were followed into young adulthood.

**Study Design:** We analyzed renin and sACE2 in serum in a prospectively followed population-based cohort at 9.9 (0.6) [mean (SD)] (*n* = 173), 11.7 (0.6) (*n* = 156), 14.8 (0.8) (*n* = 149), 18.8 (0.3) (*n* = 93), and 23.5 (0.7) (*n* = 152) years of age. Height (cm) and weight (kg) was measured and body mass index (BMI) was calculated as weight (kg)/height (m)^2^. Sex-related differences in renin levels were calculated using analysis of covariance, adjusted for age. Correlations were assessed by calculating the correlation coefficient (*R*^2^) using a multivariable linear mixed model.

**Results:** Both sexes had low renin levels up to 12 years of age. Thereafter renin levels increased more in boys than in girls. Males from the age of 15 had significantly higher levels than females (*p* < 0.001). There was a positive linear relationship between renin and sACE2 levels in male and female subjects (*p* < 0.001), and between sACE2 levels and BMI in males (*p* < 0.001).

**Conclusion:** Renin levels increase with age, are higher in men than in women since around puberty, and are correlated with sACE2 levels. Furthermore, sACE2 levels are correlated with body mass index in males. These findings indicate that high renin levels in males and females and a high BMI in males may activate pathways which increase the shedding of mACE2, with possible implications for the risk of severe coronavirus disease 2019.

**Graphical Abstract:**
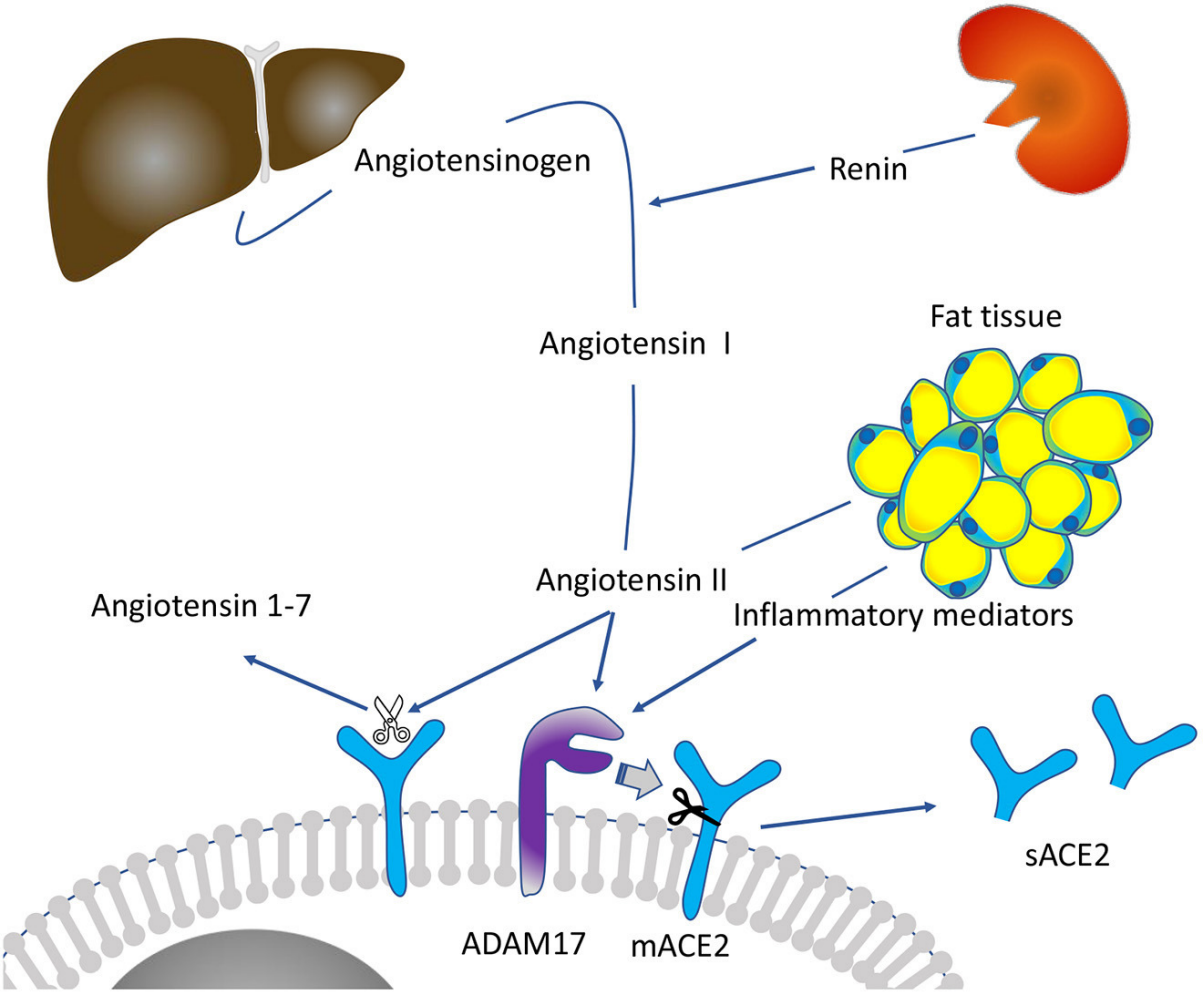
Theoretical model linking renin and excessive fat tissue to the shedding of mACE2 (membrane bound angiotensin-converting enzyme 2) and increased levels of soluble ACE2 (sACE2). ADAM17 (a disintegrin and metalloproteinase) can induce mACE2 shedding upon angiotensin II or pro-inflammatory stimuli.

## Introduction

The severe acute respiratory syndrome coronavirus-2 (SARS-CoV-2) has during 2020 caused a pandemic of Coronavirus disease 2019 (COVID-19) (Dong et al., 2020). Severe COVID-19 infection is most common among elderly men and rare among children (Grasselli et al., 2020; Suleyman et al., 2020). In young adults, the proportion with severe COVID-19 is higher among men than women (Grasselli et al., 2020). Comorbidities, including obesity, hypertension, cardiovascular disease and diabetes, have been identified as important factors for developing severe COVID-19 and COVID-19 associated mortality (Grasselli et al., 2020; Guan et al., 2020; Popkin et al., 2020). The pathophysiology behind these clinical observations is still unclear. However, a relative deficiency of membrane-bound angiotensin-converting enzyme 2 (mACE2) has been implicated as a link between cardiovascular disease, diabetes, old age, and male sex (Xie et al., 2006; Oudit and Pfeffer, 2020; Verdecchia et al., 2020; Wang et al., 2020). In line with this, it has been proposed that a pre-existing deficiency of mACE2 in the lung and other organs (which can be infected by SARS-CoV-2) (Gupta et al., 2020) may corroborate with increased risk to develop severe COVID-19 (Oudit and Pfeffer, 2020; Verdecchia et al., 2020; Wang et al., 2020).

In the basal renin-angiotensin-system (RAS) state, renin cleaves angiotensinogen into angiotensin I (ANGI), which is subsequently cleaved into ANGII by angiotensin-converting enzyme, a membrane-bound metalloproteinase highly expressed in the pulmonary circulation (Paul et al., 2006). ANGII interacts with the angiotensin II type 1 receptor subtype (AT1R), which leads to vasoconstriction and activation of pro-inflammatory and pro-fibrotic pathways (Skurk et al., 2004; Oudit and Pfeffer, 2020; Wang et al., 2020). Regulating ANGII signaling, mACE2 cleaves ANGII, generating the vasodilator angiotensin 1-7, which induces anti-inflammatory and anti-thrombotic pathways (Oudit and Pfeffer, 2020; Wang et al., 2020). These observations have led to the hypothesis that individual differences in the basal RAS signaling, which are associated with higher circulating renin levels, may lead to increased ANGII/a disintegrin and metalloproteinase-17 (ADAM-17) induced mACE2 shedding. An increased shedding could then potentially contribute to a pre-existing mACE2 deficiency with increasing age from childhood until adulthood, preferentially in men.

However, if underlying differences in the basal RAS signaling can explain why individuals with high age, male gender, and overweight/obesity, have a higher risk to develop severe COVID-19 upon SARS-CoV-2 infection is still unclear. Although previous studies have found that circulating renin levels are higher in middle-aged men compared to middle-aged women (Schunkert et al., 1997) and that circulating renin levels, and plasma renin activity correlate with plasma ANGII (Kosunen and Pakarinen, 1978; Nystrom et al., 1997), a deeper knowledge on differences in renin levels between children and young adults as well as between young men and women, is lacking.

We have shown in a recent publication that soluble ACE2 (sACE2) increases with increasing age so that young adult men have higher sACE2 compared to young women and children (Sward et al., 2020). Complementing our findings, others have shown that sACE2 levels are higher in elderly men compared to women (Kornilov et al., 2020; Sama et al., 2020) and that sACE2 levels are higher among individuals with higher body mass index (BMI) and the metabolic syndrome (Kornilov et al., 2020). However, we are lacking studies investigating the relationship between sACE2 and lung mACE2 protein levels, which is of great importance (Vaduganathan et al., 2020). Hence, it is still unclear if there are differences in the rate of ADAM-17 induced mACE2 shedding between children and young adults and between the sexes, yet such differences are potentially of importance to improve current treatment of COVID-19.

The *aim* of this observational study was to assess age-and sex differences in serum renin, and the relationship between renin and sACE2, and between renin, sACE2 and BMI in a prospectively followed population-based cohort of children, followed into young adulthood. We hypothesized that in groups with high risk to develop severe COVID-19 (adults > children and men > women), (i) renin levels would increase with age and reach higher levels in adult men compared to adult women, that (ii) renin and sACE2 levels would be positively correlated, and that (iii) renin and sACE2 would be positively correlated with BMI. Such findings would support the hypothesis that there are differences in RAS signaling between adults and children, and between men and women, and that renin levels, by affecting ANGII levels, may induce ADAM-17 activity toward mACE2.

## Subjects and Methods

### Study Population Pediatric Osteoporosis Prevention Study

The Pediatric Osteoporosis Prevention (POP) study is a prospective study that annually follow a population-based cohort of children through the nine compulsory school years, primary to evaluate development in musculoskeletal traits, fracture incidence and academic achievement in relation to physical activity. The study design has been reported in detail in previous publications (Linden et al., 2007; Detter et al., 2014; Coster et al., 2017). In short, the POP cohort includes children from four community-based and government-funded, neighboring elementary schools in the community of Bunkeflo, Malmö, Sweden. All schools were located in the same city area with similar socioeconomic status. The children were allocated to the schools depending on their residential address. The only registered difference between the four schools was that one of the schools had 40 min daily physical education (200 min/week) whereas the other three had only 60min/week (provided in 1–2 lessons).

**Figure 1:**
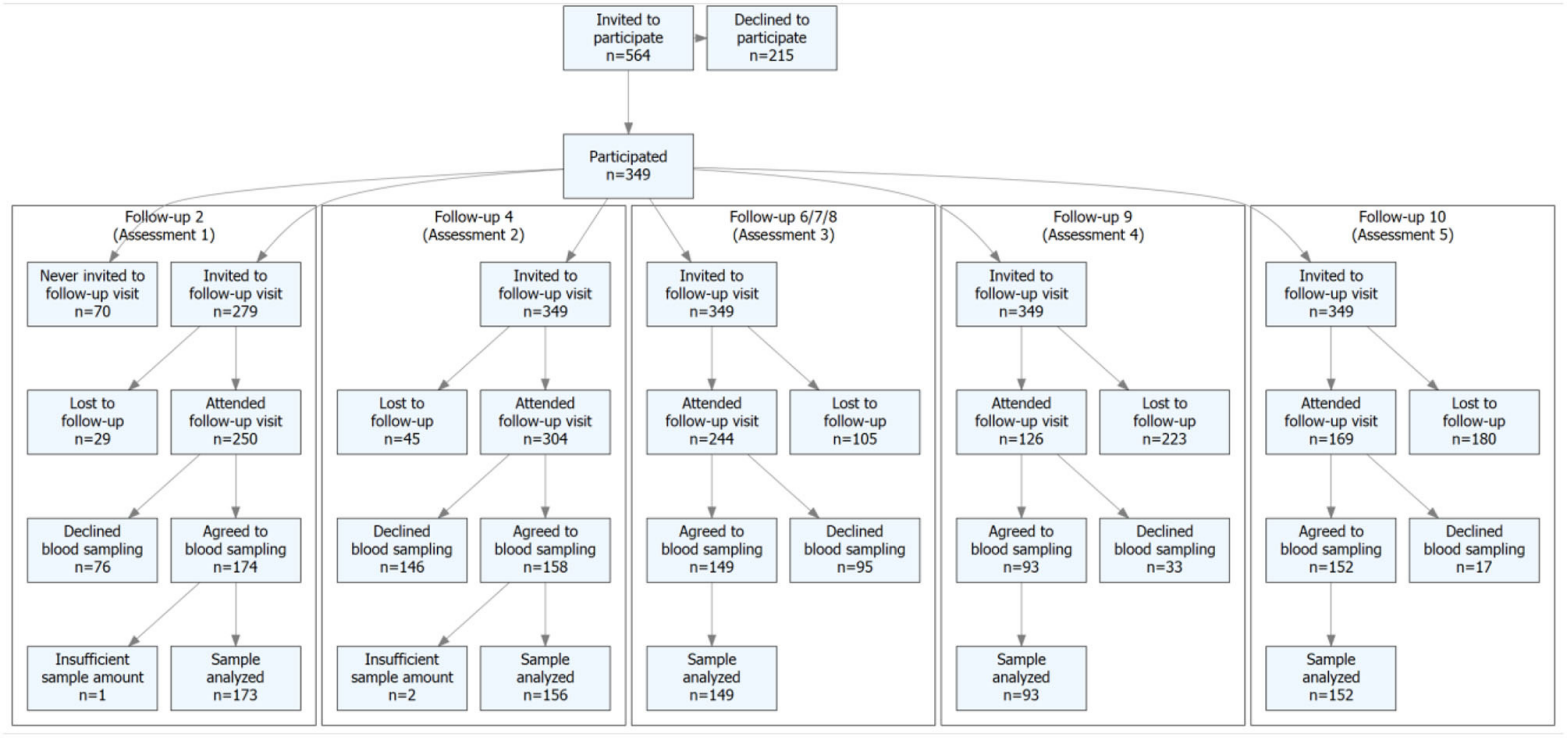
Flow-chart of subjects included in the study, and lost to follow-up.

Children who started grade 1 (1998–2000) were invited to participate at the time of school start. Of the children who agreed to participate in the study, 98% were of Caucasian ethnicity. The children were then 7.7 (0.6) years [mean (SD)] (Cronholm et al., 2020). At baseline 349 children were included in the study and the children were followed by annual measurements from baseline to grade nine, corroborating with termination of compulsory school (Cronholm et al., 2020). The POP cohort was also evaluated by measurements at mean age 19 and 24 years. Height (cm) was measured with a Holtain Stadiometer (Holtain LTD, Pembrokeshire, UK) and weight (kg) with an electric scale (Avery Berkel HL 120 Electric Scale, Avery Berkel, West Midlands, UK). Body mass index (BMI) was calculated as weight (kg)/height (m)^2^. Blood samples were collected at a mean age of 9.9 (0.6; *n* = 173), 11.7 (0.6; *n* = 156), 14.8 (0.8; *n* = 149), 18.8 (0.3; *n* = 93), and 23.5 (0.7) (*n* = 152) years of age (Sward et al., 2020) (Figure 1). Ethical approval for the POP study was obtained at the Regional Ethics Committee at Lund University, Lund, Sweden (LU 471-95, LU 486-96, and 2015/118). Written informed consent was gained from parent(s) of all children included in the study.

In a previous drop-out analysis, utilizing the general school health data register, we found at baseline similar body weight, body height and body mass index (BMI; kg/m^2^) in children who agreed or disagreed to participate at baseline (Dencker et al., 2006). A second drop-out analysis, comparing children who left blood samples at 9.9 (0.6), 11.7 (0.6), 14.8 (0.8), 18.8 (0.3), 327 and 23.5 (0.6) years of age (assessments 1–5) with those who attended at baseline but who did not donate blood is presented in Supplementary Figures 1–5.

### Laboratory Methods

Serum was prepared by letting the blood clot for 30 min at 8°C, followed by centrifugation at 1430 G for 10 min. The serum was then stored at −70°C until analysis. All samples were analyzed in the same batch, with subject's serum from the four schools randomized between the plates. Renin and sACE2 were measured using the Olink® panels (Olink Proteomics AB, Uppsala, Sweden) according to the manufacturer's instructions. The Proximity Extension Assay (PEA) technology used for the Olink protocol has been described in detail (Assarsson et al., 2014). Biomarker levels were normalized using an internal extension control and an inter-plate control, to adjust for intra- and inter-run variation. The final assay read-out is presented in Normalized Protein eXpression (NPX) values, which is an arbitrary unit on a log2-scale where a high value corresponds to a high protein expression. Detection limits, intra- and inter-assay precision data are available on manufacturer's website (www.olink.com).

## Statistical Analysis

We performed a drop-out analysis at each follow-up visit, comparing the distribution of standardized height, weight and BMI between subjects included in the study, and subjects lost during follow-up. Data from the most recent visit was used in the comparisons and distributions were compared visually. Differences in renin levels between male and female subjects were calculated using analysis of covariance, adjusted for age. Outlier observations which deviated more than 3 SD from the sex-specific mean (*n* = 8) were excluded from the analysis. To assess the relationship between renin and sACE2 we calculated the correlation coefficient (*R*^2^) using a linear mixed model taking age, sex, and repeated measures into accounts, i.e., subjects as random effects. Because that data from Olink's platform have an S-curve (sigmoid) relationship with the true protein concentration in a sample (www.olink.com), the rank-based inverse normal transformation was applied on the protein levels of renin and sACE2 prior to correlation analysis. Data is presented as mean with 95% confidence intervals (CI). The level of significance was set at *p* < 0.05, and analyses were performed using R statistical software (version 4.0.2, The R Foundation for Statistical Computing, Vienna, Austria).

## Results

Drop-out analysis showed that there were no major discernable differences when comparing the distribution of height, weight, and BMI between subjects included in the analyses and subjects lost during follow-up (Supplementary Figures 1–5). Baseline characteristics are presented in Table 1.

**Table 1:** Subject background data in relation to age and sex at repeated health examinations.

	**Baseline, Age 7.7 (SD 0.6) years**		**Age 9.9 (SD 0.6) years**		**Age 11.7 (SD 0.6) years**	
	**Boys (*n* = 191)**	**Girls (*n* = 158)**	**Boys (*n* = 92)**	**Girls (*n* = 80)**	**Boys (*n* = 88)**	**Girls (*n* = 67)**
**BACKGROUND DATA**						
Age, years (SD)	7.7 (0.6)	7.7 (0.6)	10.0 (0.6)	9.8 (0.6)	11.8 (0.6)	11.7 (0.6)
Height, cm (SD)	128.8 (6.5)	128.0 (7.0)	140.6 (6.8)	140.1 (7.3)	152.5 (8.0)	152.6 (10.0)
Weight, kg (SD)	27.7 (5.3)	27.3 (5.3)	34.3 (6.9)	34.4 (6.6)	43.4 (9.2)	43.8 (9.6)
BMI, kg/m^2^ (SD)	16.6 (2.3)	16.6 (2.4)	17.3 (2.6)	17.5 (2.7)	18.5 (2.9)	18.6 (3.3)
	**Age 14.8 (SD 0.8) years**		**Age 18.8 (SD 0.3) years**		**Age 23.5 (SD 0.7) years**	
	**Boys (*****n*** **=** **82)**	**Girls (*****n*** **=** **66)**	**Boys (*****n*** **=** **48)**	**Girls (*****n*** **=** **44)**	**Men (*****n*** **=** **75)**	**Women (*****n*** **=** **74)**
**BACKGROUND DATA**						
Age, years (SD)	14.9 (0.7)	14.7 (0.8)	18.8 (0.3)	18.8 (0.3)	23.5 (0.7)	23.5 (0.7)
Height, cm (SD)	173.2 (8.2)	165.7 (6.7)	181.8 (6.5)	168.5 (5.0)	180.6 (6.9)	168.6 (5.9)
Weight, kg (SD)	61.8 (13.2)	57.6 (11.0)	75.9 (11.9)	64.0 (10.3)	78.9 (11.8)	66.4 (12.4)
BMI, kg/m^2^ (SD)	20.5 (3.5)	20.9 (3.6)	23.0 (3.4)	22.5 (3.2)	24.1 (3.0)	23.3 (4.1)

*BMI, body mass index; SD, standard deviation*.

### Renin in Relation to Age and Sex

**Figure 2:**
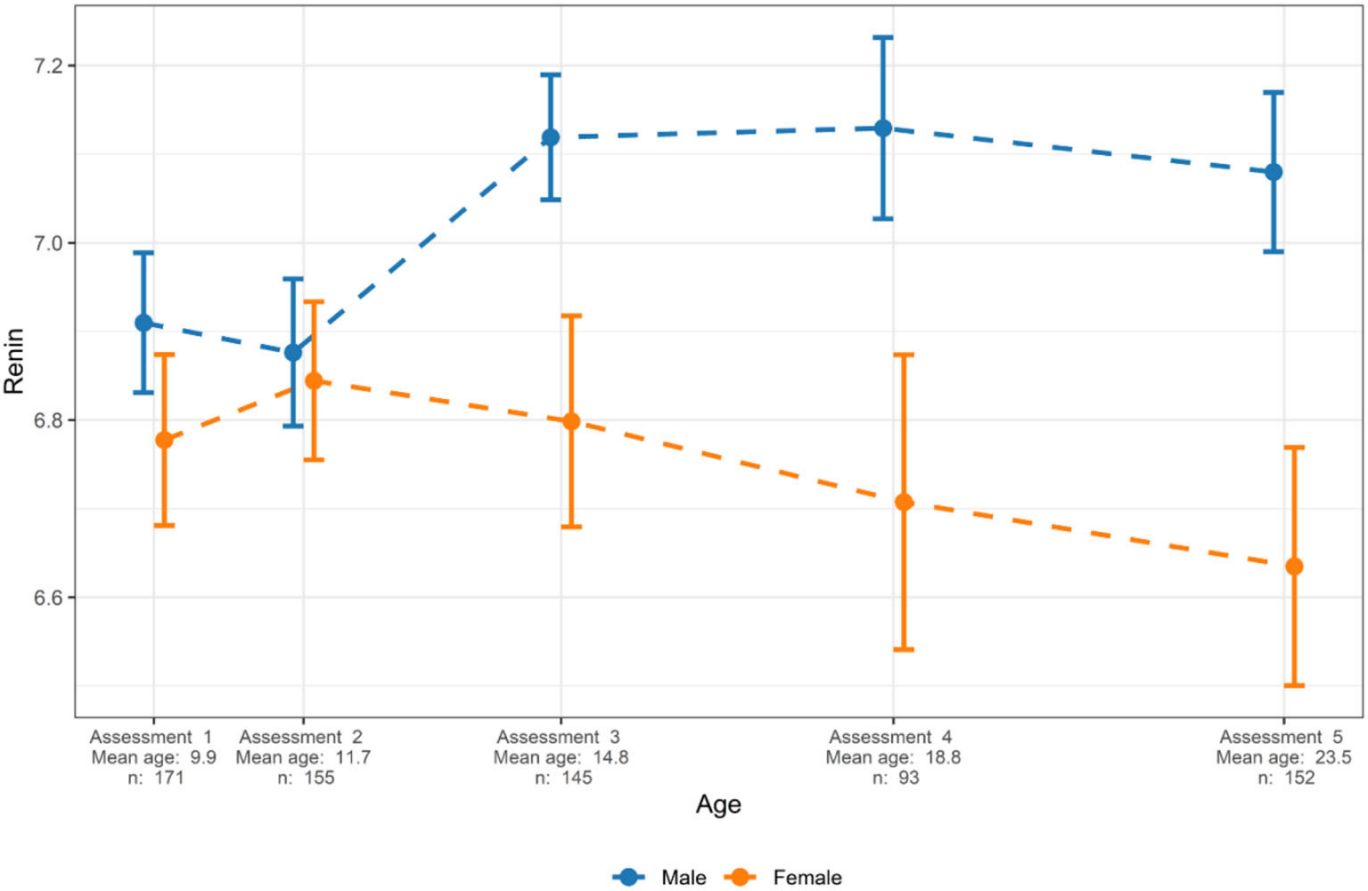
Sex-specific levels of renin in relation to age. Mean and 95% confidence intervals are presented. Renin levels are presented as normalized protein expression on a Log^2^ scale.

Sex explained 5.8% of the variance in renin (*p* = 1.6 × 10^−6^). Renin levels were similar in both sexes up until the age of 12 years. Among male subjects, renin levels increased with age and the renin levels were significantly higher among males from 15 years of age compared to males younger than 15 years of age (Figure 2). Also, whereas renin levels increased in males from age 12, they decreased in females with growth/aging and males older then 15 years of age had significantly higher renin levels compared to females (Figure 2). The mean (95% CI) renin Normalized Protein eXpression (NPX) levels were for male and female subjects at a mean age of 14.8 [7.1 (7.0–7.2) vs. 6.8 (6.7–6.9), *p* = 1.8 × 10^−4^], at mean age 18.8 [7.1 (7.0–7.2) vs. 6.7 (6.5–6.9), *p* = 4.6 × 10^−5^], and at mean age 23.5 [7.1 (7.0–7.2) vs. 6.6 (6.5–6.8), *p* = 3.0 × 10^−7^] years, corresponding to 23, 32, and 41% higher renin levels in male compared to female subjects at ages 14.8, 18.8, and 23.5 years, respectively.

### Correlations Between Renin, sACE2, and BMI

**Figure 3:**
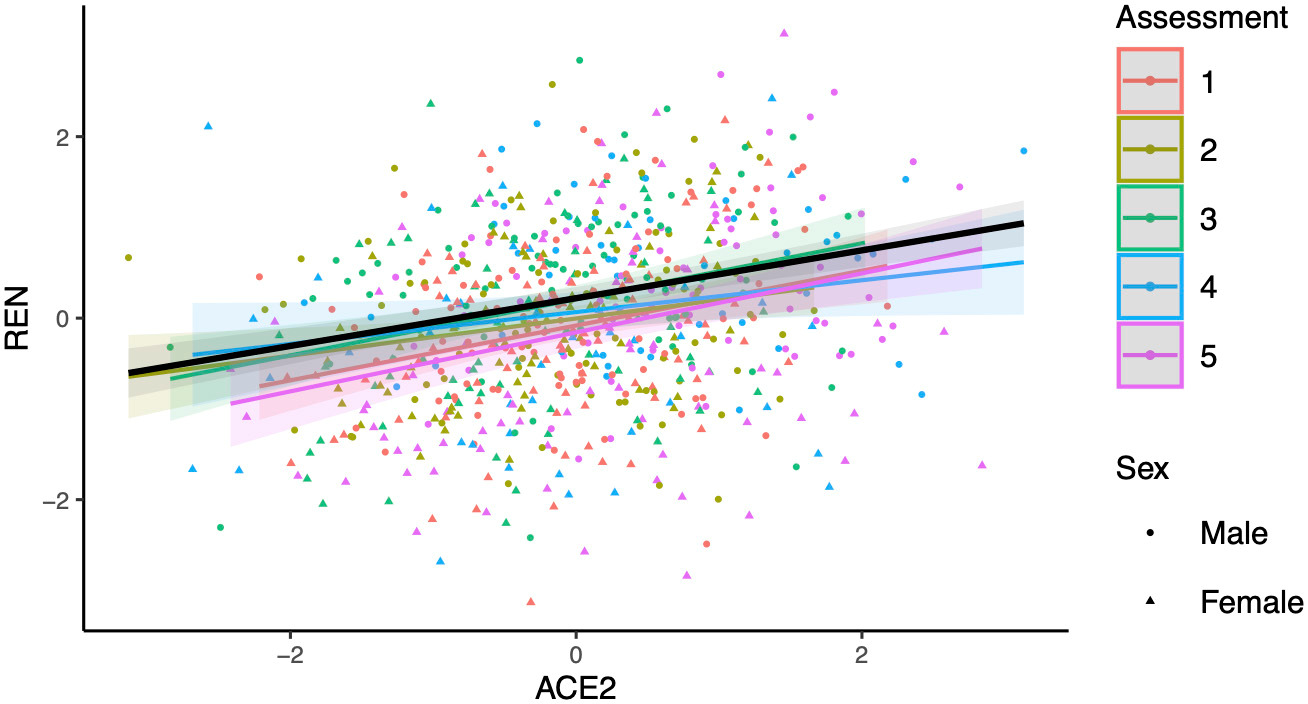
Relationship between renin (REN) and soluble angiotensin-converting enzyme 2 (ACE2). The slope (black line) and 95% confidence intervals (shaded regions) were estimated from a linear mixed model using age and sex as covariates as well subjects as random effects. The obtained *R*^2^ between renin and sACE2 is 0.072 (*p* = 9.7 × 10^−12^). Regression lines for respective groups of assessment (assessments 1–5: mean age 9.9, 11.7, 14.8, 18.8, and 23.5 years) are also shown.

No significant interaction effect for sACE2 levels and sex was found in the association with renin (*p* = 0.37). Overall, renin showed a positive correlation with sACE2 levels (*R*^2^ = 0.072, 95% CI 0.04–0.112, *p* = 9.7 × 10^−12^, Figure 3). The correlation between renin and sACE2 was also tested separately in males and females. We observed a positive correlation between renin and sACE2 in males (*R*^2^ = 0.072, 95% CI 0.031–0.128, *P* = 1.7 × 10^−6^; data not shown), and a positive correlation between renin and sACE2 in females (*R*^2^ = 0.056, 95% CI: 0.018–0.112, *P* = 2.8 × 10^−5^; data not shown).

**Figure 4:**
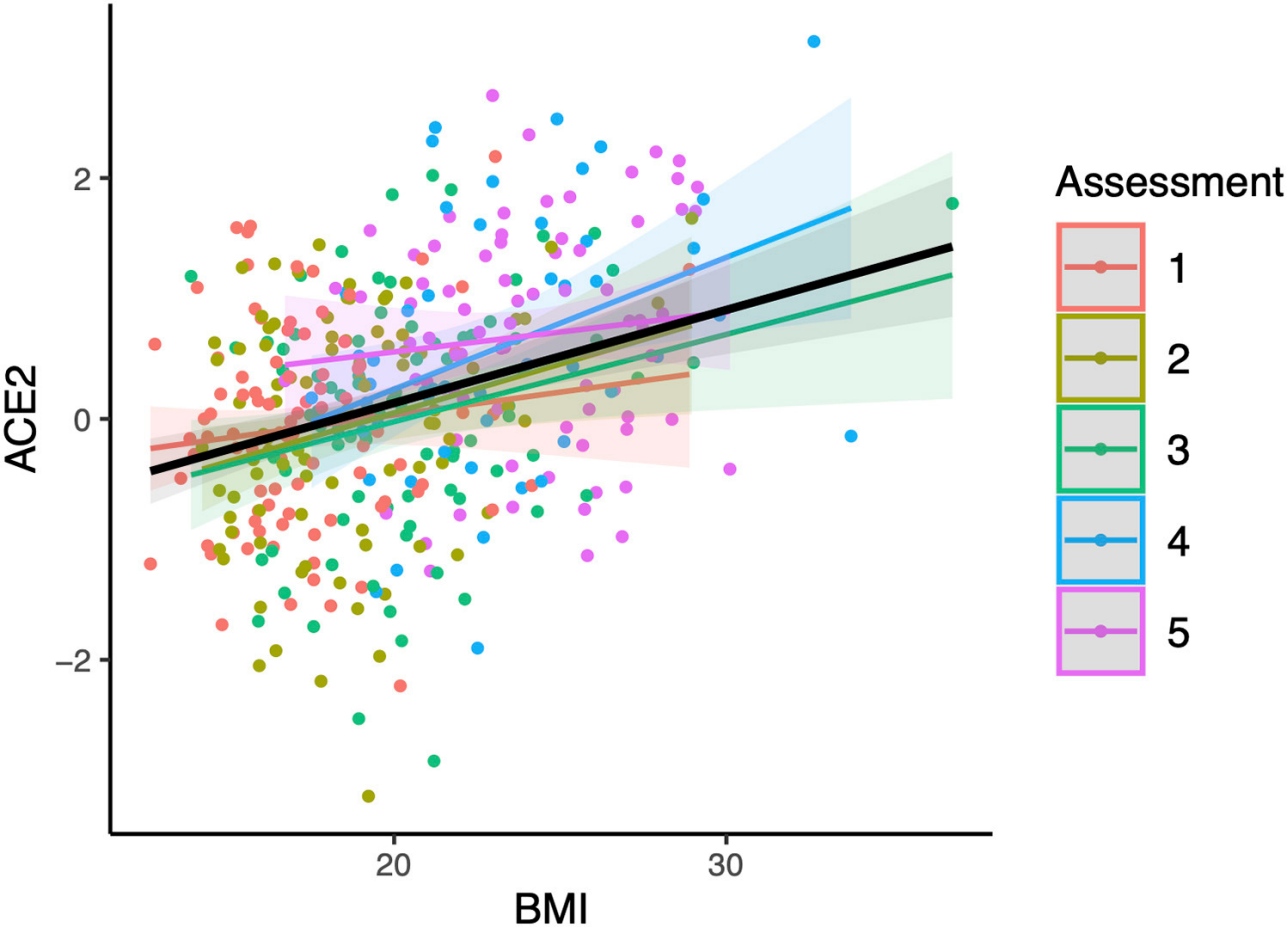
Relationship between soluble angiotensin-converting enzyme 2 (ACE2) and body mass index (BMI) in males. The slope (black line) and 95% confidence intervals (shaded regions) were estimated from a linear mixed model using age and sex as covariates as well subjects as random effects. The obtained *R*^2^ between sACE2 and BMI in males is 0.074 (*p* = 8.8 × 10^−6^). Regression lines for respective groups of assessment (assessments 1–5: mean age 9.9, 11.7, 14.8, 18.8, and 23.5 years) are also shown.

There was a significant interaction effect for sACE2 levels and sex in the association with BMI (*p* = 7.0 x 10^−8^). Therefore, the association between sACE2 levels and BMI was tested separately in males and females. We observed a significant correlation between BMI and sACE2 levels for males (*R*^2^ = 0.074, 95% CI 0.032–0.130, *p* = 8.8 × 10^−6^, Figure 4) whereas no such correlation was identified among females (*R*^2^ = 0.009, 95% CI 0–0.041, *p* = 0.14; data not shown).

Furthermore, we identified an interaction effect for renin levels and sex in the association with BMI (*p* = 0.001) but no significant correlation between renin levels and BMI in males (*R*^2^ = 0.0002, 95% CI 0–0.014, *p* = 0.79) nor in females were identified (*R*^2^ = 0.006, 95% CI 0–0.034, *p* = 0.23; data not shown).

## Discussion

The main finding of the present longitudinal study on a population-based cohort followed from childhood until young adulthood was that renin levels increased with age in boys, reaching higher levels in young men compared to young women from age 15 and onwards. This could imply underlying differences in RAS signaling between young men, children, and young women from adolescence, confirming sex-related differences in renin levels in middle-aged subjects (Schunkert et al., 1997). That renin levels in boys increase from around puberty, could suggest a positive link between renin levels, sexual maturation and increased testosterone levels. Studies in mice and rats indicate that testosterone increases pro-renin expression, and that anti-androgen treatment associates with reduced renin levels (White et al., 2019), findings which adhere with those of the present study. Also, that serum renin levels in females decrease from around puberty could be related to a downregulation of renin by estrogen. In line with these findings, estrogen therapy administered to post-menopausal women, leads to decreased plasma renin levels (White et al., 2019).

We also found that renin levels were positively correlated with sACE2 levels. This could imply that the higher sACE2 levels observed in men, by us and others (Sama et al., 2020; Sward et al., 2020), at least partly reflects increased RAS signaling/ADAM-17 activity toward mACE2 in men compared to women. Hence, underlying age- and sex differences between children and adults, and between men and women in the RAS (Fischer et al., 2002), could thereby theoretically contribute to a pre-existing mACE2 deficiency in young men compared to women and children, similar to findings in rats (Xie et al., 2006). However, this conclusion is speculative, and needs to be confirmed in other studies.

Interestingly, in the present study we showed a tendency toward decreasing levels of renin among female subjects from puberty and onwards whereas we previously showed that sACE2 tends to increase among female subjects in the same age group (Sward et al., 2020). Nevertheless, we found positive correlations between renin and sACE2 in females. Thus, although several factors (including estrogen levels) may affect the protein levels of circulating renin and mACE2 (Bukowska et al., 2017; White et al., 2019), positive correlations between serum renin and sACE2 levels in females were identified. Together this could indicate that enhanced RAS signaling/ADAM-17 dependent mACE2 shedding is reflected by increased levels of circulating sACE2.

One of the most prominent risk factors for severe COVID-19 in both children and adults is obesity. Obesity has been associated with increased risk of pneumonia, severe disease among children (Leon-Abarca, 2020; Zachariah et al., 2020), risk of hospitalization, intensive care unit admission and mortality in adults (Popkin et al., 2020). Hence, our findings of a positive correlation between sACE2 and BMI in young males may have clinical relevance which needs to be explored further.

Furthermore, our findings are in line with a previous study by Kornilov et al., where they showed that sACE2 correlated to BMI and the metabolic syndrome (Kornilov et al., 2020). Interestingly, they also found that the association between sACE2 and the metabolic syndrome was stronger among men compared to women (Kornilov et al., 2020). On the other hand, in contrast to a study by Goncalves et al. (2016), we did not find a significant correlation between renin levels and BMI. The two study cohorts are not comparable in age which could explain this difference. If the observed association between sACE2 and BMI in boys and young males is related to that RAS components (other than renin), including ANG II and inflammatory mediators can be generated in adipose tissue (Schutten et al., 2017; Delaney et al., 2018; Da Silva-Bertani et al., 2020), and thereby contribute to mACE2 shedding (Jia et al., 2009; Patel et al., 2014), needs to be explored in future studies. A complementary explanation to the association between sACE2 and BMI in males could be that ACE2 gene expression may be upregulated in obesity. In a recent publication, ACE2 gene expression was found to be higher in the lung of obese mice, and in lung epithelial cells of obese subjects, compared to non-obese subjects (Al Heialy et al., 2020).

It has been shown that RAS blockage can induce higher cardiac ACE2 mRNA and ACE2 activity in a rat model (Ferrario et al., 2005). Based on these findings, there were initial concerns that RAS blockers could increase COVID-19 risk by upregulation of the number of SARS-CoV-2 receptors. However, if these findings can be translated to the lung and to humans is unclear (Vaduganathan et al., 2020; Wysocki et al., 2020). Also, several publications have found that the treatment with ACE inhibitors or angiotensin II receptor blockers (ARBs) is not associated with the likelihood of a positive COVID-19 test, the risk of severe COVID-19 nor associated mortality (Chung et al., 2020; Lo et al., 2020; Mehta et al., 2020; Reynolds et al., 2020). Ongoing randomized trials, on the effects of RAS inhibition on outcome for SARS-CoV-2 infected patients requiring/not requiring hospital admission will provide more clarity (NCT04312009, NCT04311177).

The cumulative findings of the present study could imply higher RAS signaling and increased mACE2 shedding in males compared to females from adolescence and into young adulthood. This could be of relevance for the observed higher incidence of hypertension in young men compared to young women (Everett and Zajacova, 2015). Also, with potential importance for COVID-19, differences in mACE2 could contribute to differences in response to SARS-CoV-2 infection. The SARS-CoV-1 and SARS-CoV-2 viruses enter lung cells by binding to mACE2 (Hoffmann et al., 2020), which upon SARS-CoV-1 infection has been shown to lead to ADAM-17 induced shedding of mACE2, and increased cellular release of tumor necrosis factor (Haga et al., 2008). This may be associated with increased levels of ANGII and dysregulated RAS signaling (Kuba et al., 2005). That these events are involved in the severity of COVID-19 was implied by studies showing that ANGII levels correlate with COVID-19 lung injury in patients (Liu et al., 2020). Therefore, it was speculated that individuals with a pre-existing mACE2 deficiency, upon SARS-CoV-2 infection, may be at higher risk to develop critical mACE2 deficiency in the lung, which may lead to increased risk of acute lung injury and mortality (Verdecchia et al., 2020).

However, the role of sACE2 levels as a potential risk marker of severe COVID-19 is controversial (Rieder et al., 2020; Rojas et al., 2020). sACE2 levels are higher in males, increase with age and are associated with BMI and the metabolic syndrome (Kornilov et al., 2020; Sama et al., 2020; Sward et al., 2020), which are both described as risk factors for severe COVID-19 (Grasselli et al., 2020; Suleyman et al., 2020). Nevertheless, it has been suggested that elevated sACE2 levels may have beneficial effects on COVID-19 explained by the role of sACE2 working as a decoy receptor and thereby theoretically inhibiting the binding between SARS-COV-2 and mACE2 on host cells (Lei et al., 2020). A recent large-scaled study found that sACE2 levels were higher in patients with severe compared to non-severe COVID-19, suggesting that the relevance of this mechanism as a protective factor for severe COVID-19 may be insufficient (Filbin et al., 2020).

Study strengths include the longitudinal study design, and repeated sampling of serum in the prospectively followed population-based cohort included in the present study. Study limitations include that the cohort was only followed into young adulthood, the lack of longitudinal blood pressure data and that we cannot asses mACE2 and cell ADAM-17 activity in serum. Therefore, any conclusions with regards to ADAM-17 activity and mACE2 protein expression in the present study are speculative, but our findings highlight the need of studies designed to address age- and sex-related differences in mACE2 tissue protein levels. Although ADAM-17 is believed to be of particular importance for the shedding of mACE2 (Haga et al., 2008; Jia et al., 2009; Patel et al., 2014), other sheddases, including ADAM-10 can also cleave mACE2 (Jia et al., 2009). However, *in vitro* studies of human airway epithelial cells showed that whereas inhibition of ADAM-17 decreased the basal sACE2 release into medium, ADAM-10 inhibition did not (Jia et al., 2009), and the shedding of mACE2 by ADAM-17 after ANGII stimulation has been shown by others (Patel et al., 2014).

In conclusion, the present longitudinal study presents data showing that renin levels increase with age in children, are higher in men than in women since around puberty, and are correlated with sACE2 levels. These data could suggest that higher renin levels in men compared to women and in men compared to children, could lead to increased angiotensin II/ADAM-17 induced mACE2 shedding in men. We also identified a correlation between increasing sACE2 and BMI in males, but not in females. This could theoretically be related to increased production of RAS and inflammatory mediators in male adipose tissue, possibly combined with an adiposity-induced increase in ACE2 gene expression. In the COVID-19 context, increased RAS signaling could lead to a pre-existing mACE2 deficiency developing with increasing age, and more in men than women, with possible implications for the risk of severe COVID-19. We recommend that studies further explore this potential relationship.

## Data Availability Statement

The original contributions presented in the study are included in the article/Supplementary Material, further inquiries can be directed to the corresponding author.

## Ethics Statement

The studies involving human participants were reviewed and approved by The Regional Ethics Committee at Lund University, Lund, Sweden. Written informed consent to participate in this study was provided by the participants' legal guardian/next of kin.

## Author Contributions

The present study was designed and data analyzed by LJ, JS, PN, AE, and PS. Statistical analysis was performed by LJ and JS. PS wrote the first draft of the manuscript. All authors critically assessed the manuscript and approved the final manuscript.

## Conflict of Interest

The authors declare that the research was conducted in the absence of any commercial or financial relationships that could be construed as a potential conflict of interest.
