# Supplementary material for: Serum Renin Levels Increase With Age in Boys Resulting in Higher Renin Levels in Young Men Compared to Young Women, and Soluble Angiotensin-Converting Enzyme 2 Correlates With Renin and Body Mass Index
Source: Front Physiol. 2021 Jan 15;11:622179. doi: 10.3389/fphys.2020.622179 (PMC7844344; doi:10.3389/fphys.2020.622179)
Supplement: Supplementary file 1 [file Data_Sheet_1.DOCX]

**Supplementary material**

**Serum renin levels increase with age in boys resulting in higher renin levels in young men compared to young women, and soluble angiotensin-converting enzyme 2 correlates with renin and body mass index**

Lars Jehpsson^1^*, Jiangming Sun^2^*, Peter M Nilsson^3^, Andreas Edsfeldt^2,4,5^ and Per Swärd^1^

^1^ Clinical and Molecular Osteoporosis Research Unit, Departments of Orthopedics and Clinical Sciences, Skåne University Hospital, Lund University, Malmö, Sweden

^2^ Department of Cardiovascular Research-Translational Studies and Cardiology, Skåne University Hospital, Lund University, Sweden

^3^ Internal Medicine - Epidemiology, Department of Clinical Sciences, Lund University, Skane University Hospital, Malmö, Sweden

^4^ Wallenberg Center for Molecular Medicine, Lund University, Sweden

^5^ Dept. of Cardiology, Skåne University Hospital, Malmö, Sweden

*These authors contributed equally to the manuscript

Corresponding author: Per Swärd MD. PhD. Clinical and Molecular Osteoporosis Research Unit, Department of Orthopedics, Skåne University Hospital, SE - 205 02 Malmö, Sweden,

Tel +4640331000; Fax+4640336200; E-mail: [per.sward@skane.se](mailto:per.sward@skane.se)

**Table of Contents**

Supplementary figure 1 3

Supplementary figure 2 4

Supplementary figure 3 5

Supplementary figure 4 6

Supplementary figure 5 7


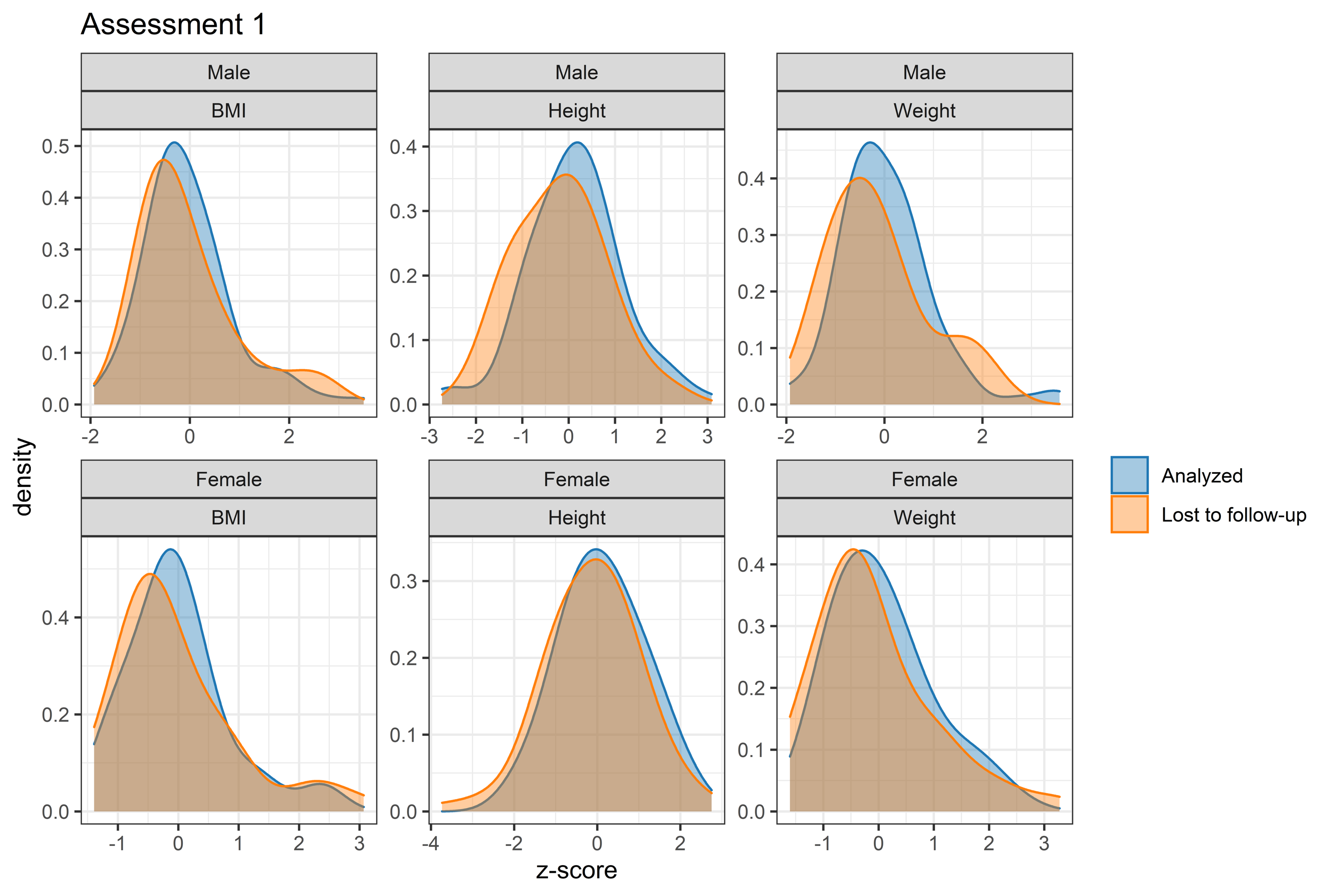


**Supplementary figure 1.** Drop-out analysis at assessment 1, at mean (SD) age 9.9 (0.6) years, comparing the distribution of standardized height, weight and BMI between subjects included in the study, and subjects lost to follow-up. Data from the most recent visit was used in the comparison.


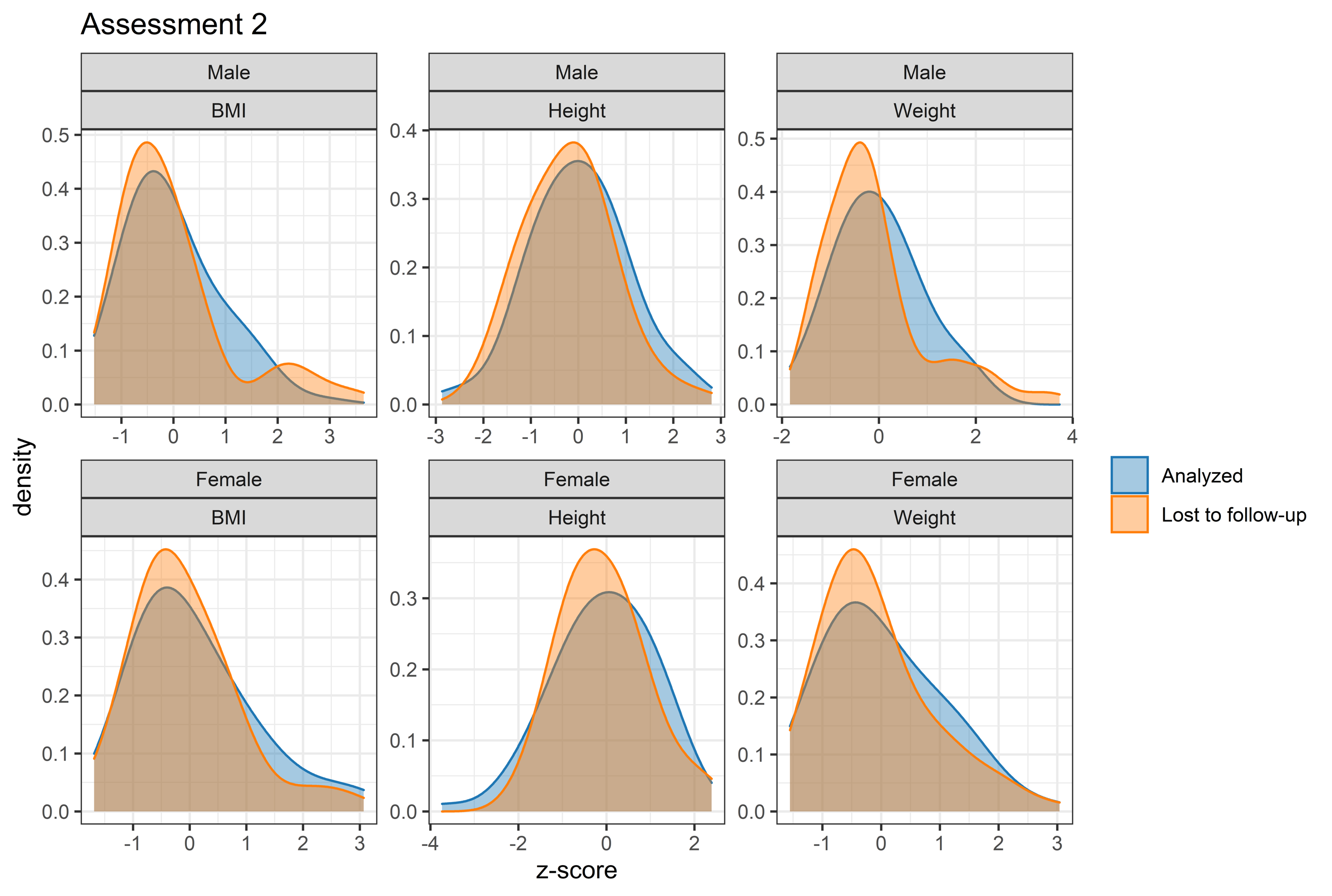


**Supplementary figure 2.** Drop-out analysis at assessment 2, at mean (SD) age 11.9 (0.6) years, comparing the distribution of standardized height, weight and BMI between subjects included in the study, and subjects lost to follow-up. Data from the most recent visit was used in the comparison.


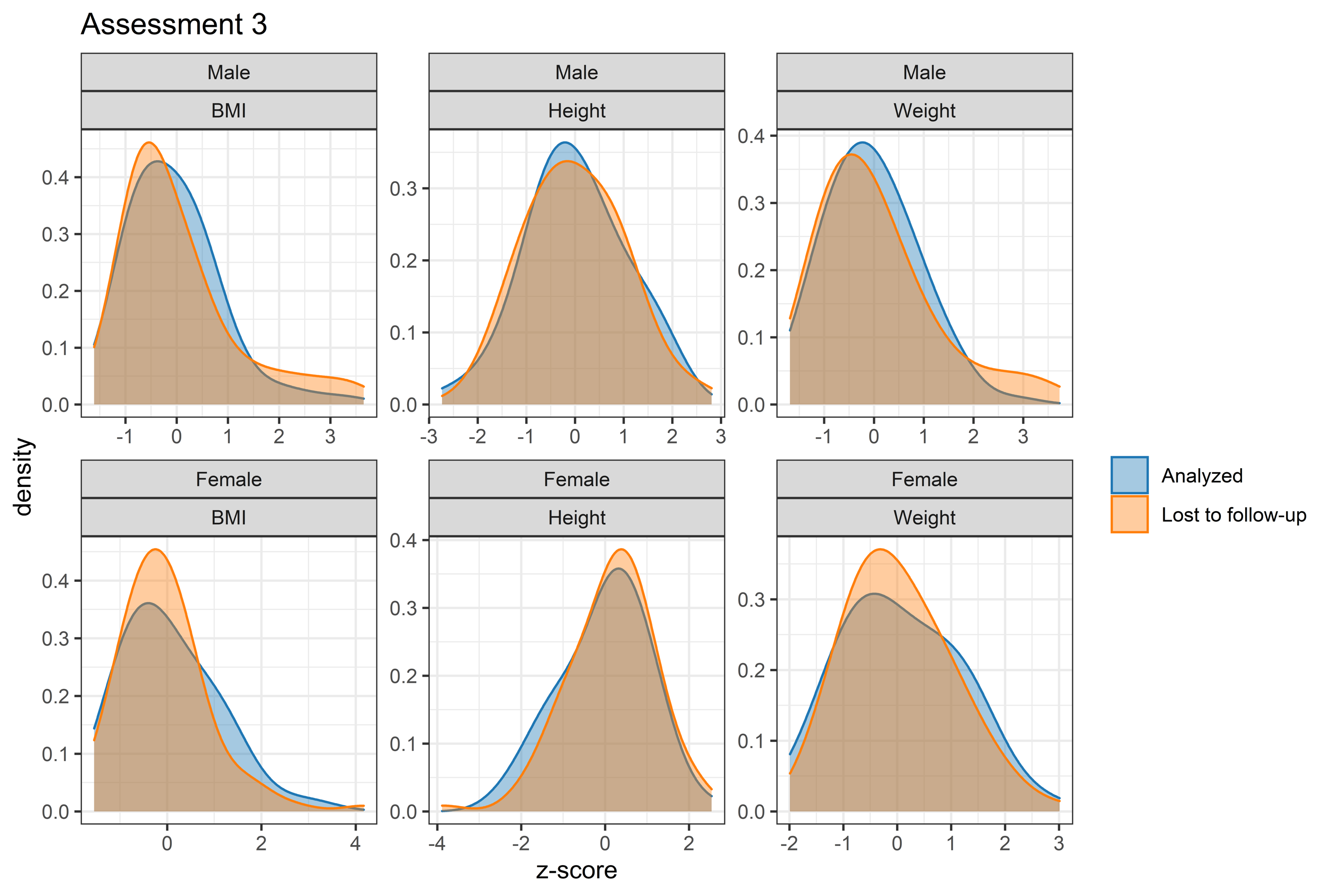


**Supplementary figure 3.** Drop-out analysis at assessment 3, at mean (SD) age 14.8 (0.8) years, comparing the distribution of standardized height, weight and BMI between subjects included in the study, and subjects lost to follow-up. Data from the most recent visit was used in the comparison.


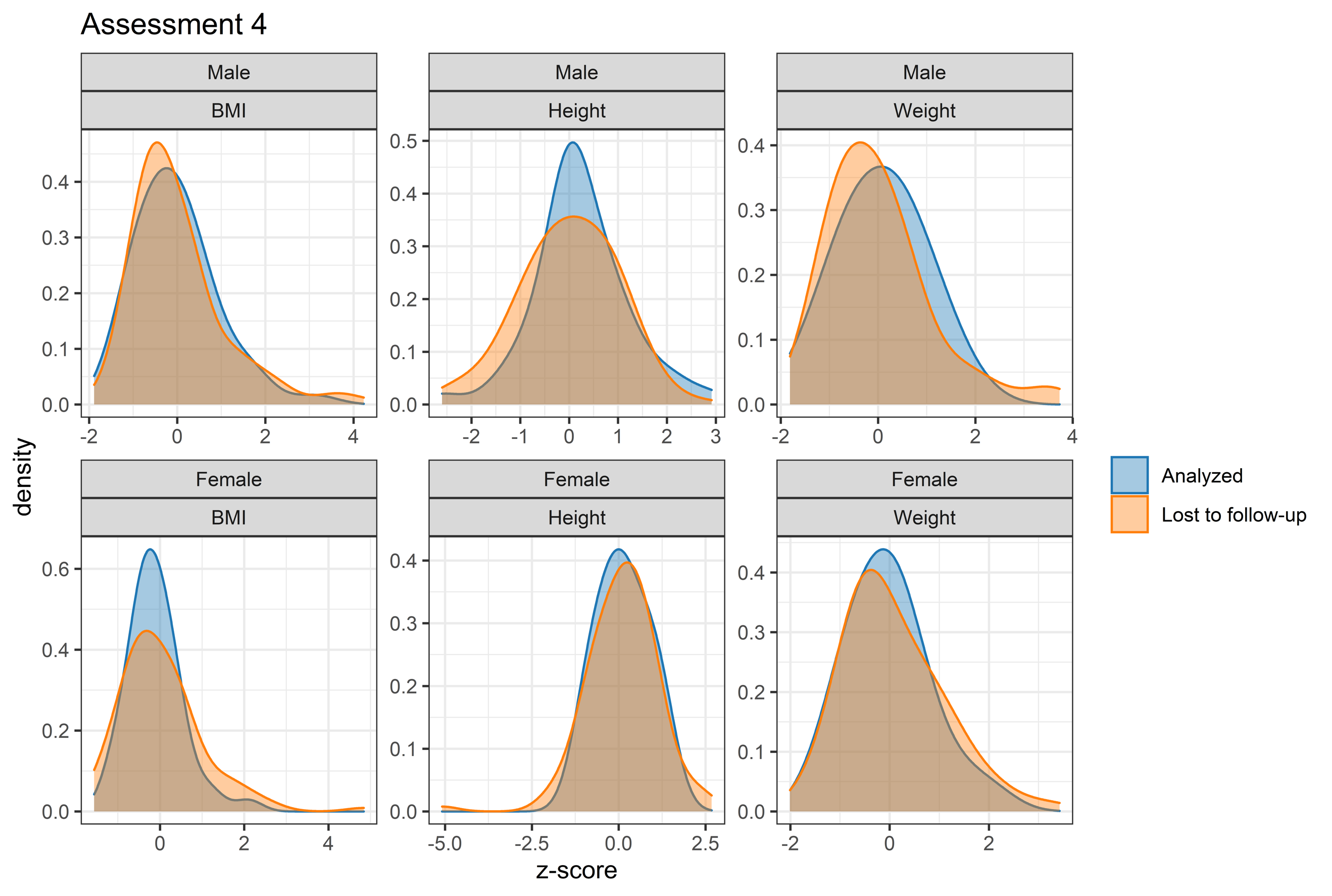


**Supplementary figure 4.** Drop-out analysis at assessment 4, at mean (SD) age 18.8 (0.3) years, comparing the distribution of standardized height, weight and BMI between subjects included in the study, and subjects lost to follow-up. Data from the most recent visit was used in the comparison.


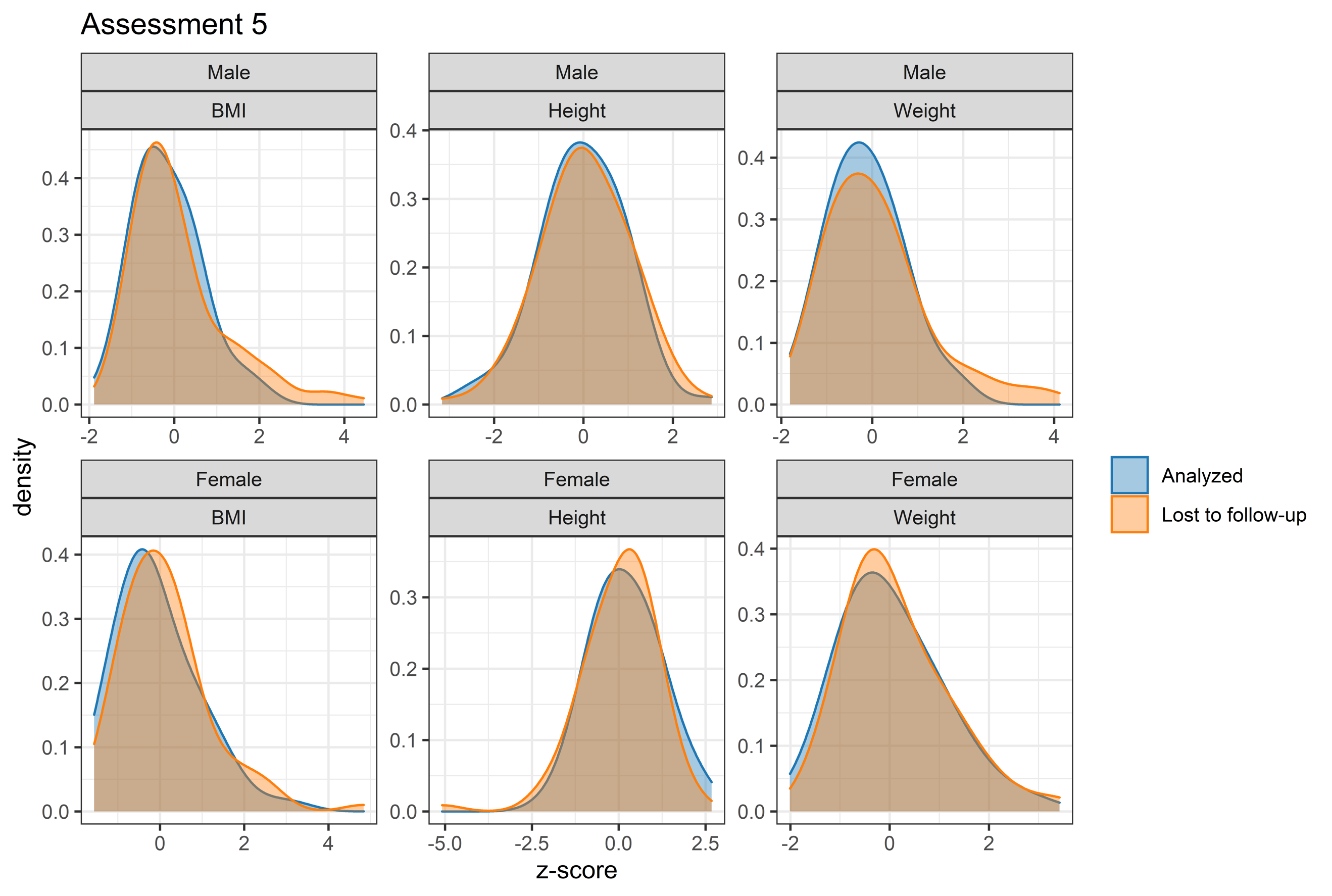


**Supplementary figure 5.** Drop-out analysis at assessments 5, at mean (SD) age 23.5 (0.7) years, comparing the distribution of standardized height, weight and BMI between subjects included in the study, and subjects lost to follow-up. Data from the most recent visit was used in the comparison.
